# Apoplexy

**Published:** 1842-07-16

**Authors:** 


### *Apoplexy.*

Three cases were treated in all, one woman, one white and one black man.

S. K., æt. 49, of a short stout frame, robust constitution, and very much inclined to obesity, entered the ward in a state of intoxication. On the following day she complained of vertigo, and stated that the period for menstruation had now arrived, but that two periods had passed without its appearance. She was directed to take a purge in the morning, and a hip bath in the evening, with abstemious diet. The next morning she was suddenly seized with giddiness while walking, and almost immediately fell down in a state of insensibility. She soon recovered her consciousness, however, but complained of a violent pain in her head. This was relieved after a few days by a full bleeding, leeching to the neck of the uterus, and tartrate of antimony and potassa in large doses. At the end of ten days the severity of of the symptoms left her, but incontinence of urine had supervened, which was not relieved by the application of blisters to the sacrum, and the internal use of the tincture of cantharides, although strangury was twice produced.

The other cases of apoplexy occurred in old men, and were of long standing. Hemiplegia was the effect of it in both cases. They entered the Hospital to seek an asylum, and with no expectation of relief from their complaints.

